# Study Protocol for the Daasanach Human Biology Project: A Longitudinal Study of the Impact of Drinking Water Salinity and Heat Stress on Water Insecurity, Hydration, Energetics, Hypertension, and Kidney Function in Northern Kenya

**DOI:** 10.1002/ajhb.70343

**Published:** 2026-10-12

**Authors:** Alfredo J. Rojas, Emmanuel Ndiema, Rosemary Nzunza, Michael Hunter, Jeff M. Sands, William Farquhar, W. Larry Kenney, Kedir Teji Roba, Amanda McGrosky, Matthew Douglass, Herman Pontzer, Asher Y. Rosinger

**Affiliations:** ^1^ Department of Biobehavioral Health Pennsylvania State University University Park Pennsylvania USA; ^2^ Department of Earth Sciences National Museums of Kenya Nairobi Kenya; ^3^ Center for Virus Research Kenya Medical Research Institute (KEMRI) Nairobi Kenya; ^4^ Department of Human Development and Family Studies Pennsylvania State University University Park Pennsylvania USA; ^5^ School of Medicine Emory University Atlanta Georgia USA; ^6^ Department of Kinesiology and Applied Physiology University of Delaware Newark Delaware USA; ^7^ Department of Kinesiology Pennsylvania State University University Park Pennsylvania USA; ^8^ Haramaya University, College of Health and Medical Sciences Haramaya Ethiopia; ^9^ Department of Biology Elon University Elon North Carolina USA; ^10^ College of Agricultural Sciences and Natural Resources University of Nebraska‐Lincoln Lincoln Nebraska USA; ^11^ Department of Evolutionary Anthropology Duke University Durham North Carolina USA; ^12^ Global Health Institute Duke University Durham North Carolina USA; ^13^ Department of Anthropology Pennsylvania State University University Park Pennsylvania USA

**Keywords:** chronic kidney disease, heat, hypertension, Kenya, water salinity

## Abstract

**Introduction:**

Increasing salinity of groundwater is a global environmental health threat that has been overlooked in human biology and may increase the risk of water insecurity, dehydration, chronic kidney disease (CKD), and hypertension in humans. However, no health standards exist for salinity levels in drinking water. This protocol paper describes the Daasanach Human Biology Project, which is testing how water salinity exposure and heat stress impact hydration, kidney disease, and blood pressure among Daasanach pastoralists in a hot‐arid environment in northern Kenya.

**Methods/design:**

This study expands the Daasanach Human Biology Project, an existing longitudinal study, to recruit 600 adults across approximately 450 households in 12 communities in Illeret Sub‐County, Marsabit County, Kenya across six study waves. Data collection will occur seasonally during the wet (March–April) and dry (June–July) seasons. Household survey, anthropometric, dietary, doubly labeled water, point‐of‐care, lab‐based biomarker, and water quality data will be collected to assess the impact of water salinity, heat stress, and other relevant exposures on health. Multi‐level regression will be used to account for the longitudinal and nested nature of the data to determine how water salinity coupled with environmental exposures like heat impact hydration, kidney, and cardiometabolic function, and hypertension among Daasanach pastoralists.

**Discussion:**

Exploring how water salinity in hot environments impacts human health can help uncover the environmental and social pathways to CKD and cardiovascular diseases facing populations around the world dealing with freshwater salinization. The data generated will provide dose‐specific health standards for sodium‐chloride in water.

## 1 Introduction

The field of human biology has a long history of examining how pastoralism, the practice of raising and producing livestock, especially as a subsistence strategy across the African continent, has influenced adaptations, behaviors, and health (Leonard 2025; Little 1989). African pastoralists have been the focus of several ethnographic, ecological, demographic, and biobehavioral health studies (Leslie and Fry 1989; McCabe 2004; Riesman 1998). Subsisting in patchy ecosystems where rainfall is highly seasonal is relevant to pastoralists' mobility patterns, demographic characteristics, as well as their nutritional and biological health (Galvin 2009; Little 1989). Understanding how pastoralists adapt to and respond to environmental constraints has characterized several research projects within the field of human biology, like those among Ariaal, Pokot, Rendille, Samburu, and Turkana of northern Kenya (Fujita et al. 2025; Galvin 1992; Nathan et al. 1996; Pike et al. 2010; Sayre et al. 2019; Straight et al. 2025).

One important area of these studies is understanding the intersection of environmental health and biology of pastoralists, especially as environmental and demographic transitions become more commonplace (Leonard 2025). The Turkana, for example, are nomadic pastoralists that inhabit an extremely hot and dry environment in northwestern Kenya. Traditionally nomadic lifestyles in rural areas have been undergoing change as more individuals move to highly urbanized contexts, introducing new cardiometabolic health challenges (Lea et al. 2020, 2021). Moreover, this research has uncovered genetic adaptations that have allowed Turkana to subsist in water‐stressed areas over time (Lea et al. 2025). These insights help inform other studies on pastoralists that analyze health outcomes like dehydration in the face of extreme heat (Bethancourt et al. 2021). Limited water resources pose health risks for pastoralists in arid environments, especially as extreme weather events further exacerbate water scarcity. As pastoralists are more likely to face extreme heat, drought, and other exposures given their often water‐stressed environments, there is an urgent need to understand how these exposures affect their health and biology.

One under‐explored environmental health issue for pastoralists is the increasing salinity of groundwater, or freshwater salinization, which is a risk factor for hundreds of millions of adults around the world (Khan et al. 2014; Rivett et al. 2019; Scheelbeek et al. 2017; Watts et al. 2021). Water salinity refers to the overall concentration of salt in a water source. The accumulation of salts in freshwater is a growing problem globally, particularly in semi‐arid African regions, U.S. coastal sites suffering from saltwater intrusion to groundwater, and in areas reliant on lake water where runoff from salting roads is leading to increased concentrations (Comte et al. 2016; Kaushal et al. 2021; Rivett et al. 2019, 2020). Unlike dietary salt, health standards do not exist for maximum salinity levels in water, only that palatability of water is unsatisfactory above 200–250 mg/L (Agency 1986; World Health Organization 2008). Water salinization may compound environmental population health differences and may lead to higher rates of dehydration (Brooks et al. 2017), chronic kidney disease (CKD) (Norton et al. 2016; Said and Hernandez 2015), and hypertension (Mills et al. 2016). Emerging evidence suggests water salinization adversely affects these health outcomes, yet salinization has received little attention.

This paper describes the protocol for a multi‐year study funded by the National Institute of Environmental Health Sciences (NIEHS, R01ES035402), which expands the Daasanach Human Biology Project, an ongoing longitudinal study. This project investigates how water salinity in an extremely hot context in northern Kenya affects hydration, kidney function, and blood pressure among Daasanach pastoralists. Importantly, this work intersects with several key issues in human biology, including water insecurity, hydration, adaptation, the effects of extreme weather events, and environmental and lifestyle changes—all of which have recently been highlighted as emerging themes in human biology (Hoke and Schell 2020; Niclou and Sarma 2024; Pisor and Jones 2021; Rosinger 2023; Rosinger and Brewis 2020).

### 1.1 Water Salinity as an Exposure

Water salinity increases water insecurity, defined as insufficient and uncertain access to adequate water for a lifestyle that is active, healthy, and culturally acceptable (Benneyworth et al. 2016; Jepson et al. 2017). Long‐standing problems like lack of access to safe drinking water due to inadequate resources contribute to the social determinants of health. Water salinity is one of the biggest risk factors for global water insecurity (Damania et al. 2019). Climatic and anthropogenic processes are further exacerbating problems with water salinity both domestically (Jasechko and Perrone 2020) and globally (Gleick 2014, 2016). The share of the global population experiencing extreme heat (mean temperatures > 29°C) is expected to reach 1–3 billion people by 2070 (Mekonnen and Hoekstra 2016; WHO and UNICEF 2017; Xu et al. 2020). When severe water scarcity is coupled with ambient heat stress, water salinization is exacerbated and may create dangerous co‐exposures. Recent work has found increasing evidence of a detrimental effect of drinking water sodium on blood pressure (BP), risk of pre‐eclampsia, hypertension and gestational hypertension, and potentially kidney damage (Khan et al. 2014; Naser et al. 2017, 2019; Rakib et al. 2019; Rosinger et al. 2021, 2025; Scheelbeek et al. 2017; Shammi et al. 2019; Talukder et al. 2016).

Kidney injuries and CKD are rising in prevalence worldwide, especially in low‐income countries, affecting 8%–16% of adults globally (Clark et al. 2016; Jha et al. 2013). Screening, population awareness, and surveillance for CKD are low (Fabian et al. 2022; Saran et al. 2017). Drinking water salinity may be an invisible environmental exposure increasing kidney damage and hypertension (Rosinger and Young 2020; Shammi et al. 2019). Prior work found that regular high salt intake in water was associated with an increase in sodium excretion and increased protein excretion, indicating progressive kidney dysfunction (Naser et al. 2020). Yet the evidence linking drinking water salinity to kidney damage is scarce. More work supports a connection between raised systolic and diastolic blood pressure (BP) and risk of hypertension (Khan et al. 2014; Naser et al. 2019; Scheelbeek et al. 2017; Shammi et al. 2019). Also, little is known about how water salinity affects health when coupled with heat stress. In hot settings, physiological water needs increase, yet water scarcity and salinity may make it harder to meet these needs, leading to dehydration and water insecurity. Repeated chronic dehydration in extreme heat causes kidney injury and permanent damage (Glaser et al. 2016; Johnson et al. 2019). Thus, in hot areas with increasing water salinity, it is critical to assess these co‐exposures and screen for CKD.

A critical gap exists connecting the potential impacts of water salinity levels to risk of dehydration, water insecurity, kidney damage, and hypertension. The pathways through which hypertension and CKD develop are often silent and occur over a lifetime, and salt intake is a key risk factor (Babcock et al. 2020; Edwards and Farquhar 2015; Farquhar et al. 2015). Globally, the prevalence of hypertension is increasing (Forouzanfar et al. 2017) and is the leading risk factor of death globally, accounting for 10.4 million deaths annually (Stanaway et al. 2018) in particular for cardiovascular disease and CKD (Jha et al. 2013; Naghavi et al. 2017). Salinization and extreme heat will likely interact to exacerbate hypertension‐ and CKD‐related mortality (Vicedo‐Cabrera et al. 2021). Without specific health standards, salt in drinking water can have negative effects on chronic disease risk factors by being a hidden source of sodium and potentially a key contributing factor to growing CKD rates (Cole 1949; Elliott and Alexander 1961). An integration of water salinity in human biology and CKD research is critical since this is an emerging problem as seen in several regions in the US (Hintz et al. 2022; Kaushal et al. 2005, 2021). Testing how water salinity in hot settings affects health will improve our understanding of environmental and social pathways to the development of CKD and hypertension as well as help identify populations most at risk to unmask the true burden of disease (Barraclough et al. 2017; Johnson et al. 2019).

### 1.2 Study Objectives

The overall objectives of the Daasanach Human Biology Project are to expand the longitudinal study and analyze the impact of drinking water salinity on hydration, kidney function, and blood pressure among Daasanach pastoralists in northeastern Kenya. This objective will help researchers understand how the social and physical environments get embodied in health outcomes (Krieger 2005). Previous work has shown that Daasanach identify water salinity, water access, and food insecurity as key issues affecting health and well‐being (Bethancourt et al. 2021, 2023; Ford et al. 2022). Daasanach communities rely on various water sources like hand‐dug wells, standpipes, water from Lake Turkana, and treated, desalinated water. Communities, therefore, vary in their exposure to levels of water salinity. This project utilizes a longitudinal research design whereby communities and individuals are visited during the dry and rainy seasons twice per year over three years. Using household surveys, biomarker collection, point‐of‐care (POC) devices, water quality testing, measures of heat stress, and a natural experiment through the assessment of desalinated water, this project employes a novel and community‐engaged approach to understand the impact of water salinity on human health.

This study centers around three specific aims (see Figure 1):

**FIGURE 1:**
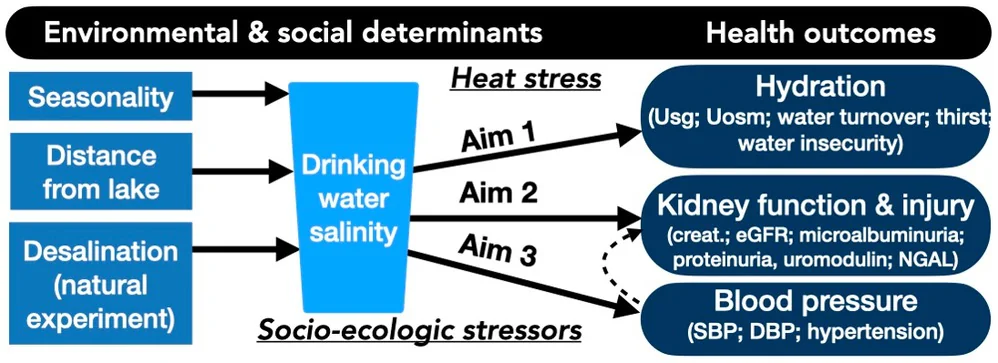
Conceptual framework illustrating specific aims.

Aim 1: To identify how variation in drinking water salinity affects hydration status, water turnover, thirst, and water insecurity.

Aim 2: To test how drinking water salinity affects kidney health over time.

Aim 3: To test how drinking water salinity affects systolic and diastolic BP longitudinally, and whether these BP changes affect kidney health.

Across aims, we will analyze how biological sex (male/female), reproductive status (non‐pregnant/pregnant/lactating), and age differences (18–45 years/45+ years) influence outcomes across the life course.

### 1.3 Study Hypotheses

Hypothesis 1 *Water salinity will be higher across water sources in the dry season and increase dehydration, water turnover* (*i.e., daily total water throughput in the body*), *water insecurity, and thirst*.

Hypothesis 2 *Higher water salinity will negatively affect kidney function* (*higher serum creatinine and lower estimated glomerular filtration rate* [*eGFR*]) *or lead to kidney injury* (*albumin‐to‐creatinine ratio* [*ACR*] ≥ 30 mg/g, *proteinuria, uromodulin (*Houser et al. 2021; Schaeffer et al. 2021; Zhang et al. 2018), *and urine Neutrophil gelatinase‐associated lipocalin* [*NGAL*]) (Barnett et al. 2022).

Hypothesis 3 *Higher water salinity will increase systolic and diastolic BP and risk of hypertension* (Scheelbeek et al. 2017).

Hypothesis 4 *Hypertension will mediate the association between salinity and kidney damage*.

## 2 Methods and Analysis

### 2.1 Setting

The Daasanach Human Biology Project takes place in Marsabit County, Kenya, a region located in the north of the country and east of Lake Turkana, the world's largest permanent desert lake (Figure 2). Daasanach are an indigenous group of semi‐nomadic pastoralists who inhabit this area (4.314°N, 36.227°E), which is in the top 1% hottest land areas in the world (mean annual temperature is 29.2°C) (Passey et al. 2010).

**FIGURE 2:**
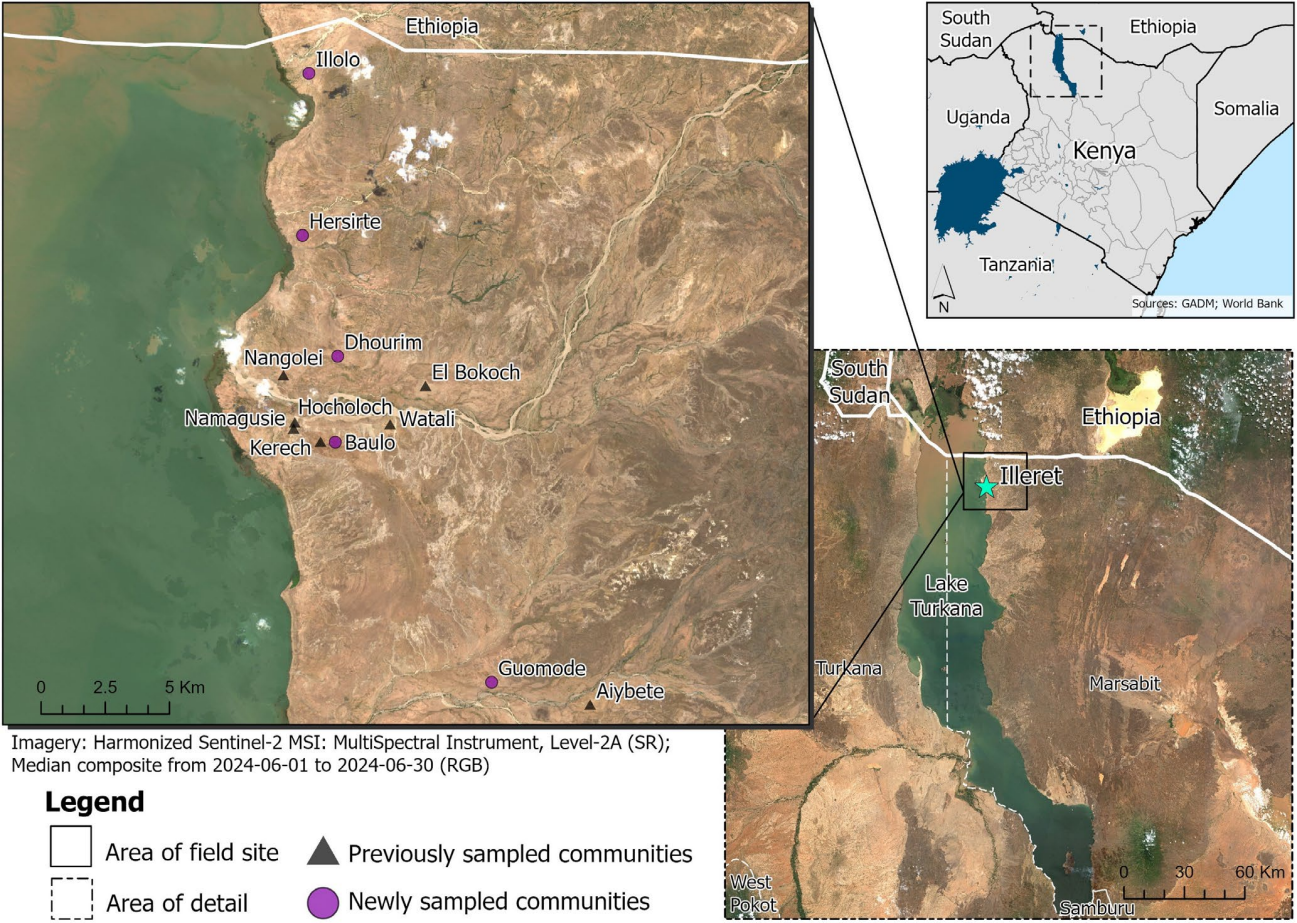
Field site and community locations of the Daasanach human biology project.

Daasanach in northern Kenya number ~19,000 across 26 communities (Kenya National Bureau of Statistics (KNBS) 2019), while a further ~46,000 Daasanach live along the Omo River in southern Ethiopia (Gebre 2012). Like many pastoralist groups, Daasanach rely on cattle and milk from livestock as a major source of calories, but historically they have had limited access to market and agricultural goods (Almagor 1978). As a population without many lifestyle risk factors for kidney disease or hypertension present in the US, this setting will help untangle the effects of water salinity from other risk factors. Daasanach are tall and lean (mean BMI of 18.2), physically active, with low rates of diabetes—key protective factors against CKD (Stengel et al. 2003) and hypertension (Chobanian et al. 2003; Pescatello et al. 2004). They consume high quantities of milk, which may be protective of hypertension (Houston and Harper 2008).

Yet, the prevalence of hypertension and poor kidney function among the Daasanach is disproportionately high given their lifestyle and health compared to Kenyan and US populations (Table 1). The variation in distance of communities from the main town captures variation in water source use and access, diet and lifestyle, and environmental exposures, which also helps compare to other ongoing work with Turkana pastoralists (Lea et al. 2021). Our work has found that Daasanach experience extremely high levels of food insecurity and water insecurity, which worsen during droughts (Rosinger et al. 2026).

**TABLE 1:** Comparison of health metrics of Daasanach sub‐population with Kenyan and U.S. populations.

Mean	Daasanach^a^	Kenya	US
BMI	18.2	23.5 Mkuu et al. (2021)	29.3 Fryar et al. (2018)
Physically inactive	~2.5% (all elderly)	7.7% Gichu et al. (2018)	25.3% Centers for Disease Control and Prevention (2023)
Diabetes	1.8%	2.4% Mohamed, Mwangi, et al. (2018)	14.7% Prevention (2023)
BP ≥ 130|80	46.8%	No data	45.4% Ostchega et al. (2020)*
BP ≥ 140|90	19.7%	24.5% Mohamed, Mutua, et al. (2018)	—
ACR ≥ 30 mg/g	39.2%	No data	10.2% Johansen et al. (2022)
CKD	No data	9.7% Bikbov et al. (2020)	14% Johansen et al. (2022)

Abbreviations: ACR, albumin‐to‐creatinine ratio; BMI, body mass index; BP, blood pressure; CKD, chronic kidney disease; HTN, hypertension.

^a^ Data from our Daasanach longitudinal study; *In US, HTN also defined as taking BP‐lowering medication.

The Daasanach Human Biology Project, which has previously been called the Daasanach Health and Life History Project, has been working with Daasanach communities to test water quality since 2017. In 2019, we began a longitudinal study to assess long‐term perspectives on water and food insecurity, socio ecological stressors, hydration, nutrition, energetics, and health with 7 of the 26 communities in the area (Baker et al. 2025; Bethancourt et al. 2021, 2023; Ford et al. 2022; McGrosky et al. 2025, 2024; Roba et al. 2025; Rosinger et al. 2021, 2022, 2025, 2024; Sadhir et al. 2025; Swanson, Bethancourt, et al. 2023; Swanson, Nzunza, et al. 2023). That longitudinal study collected previous study waves during the dry season in June–July of 2019, 2022, 2023, and 2024 (Figure 3). Additionally, a subsample survey wave among previously participating adults (*n* = 176) was conducted during the rainy in April 2025 to explore seasonal variation in exposures and outcomes.

**FIGURE 3:**
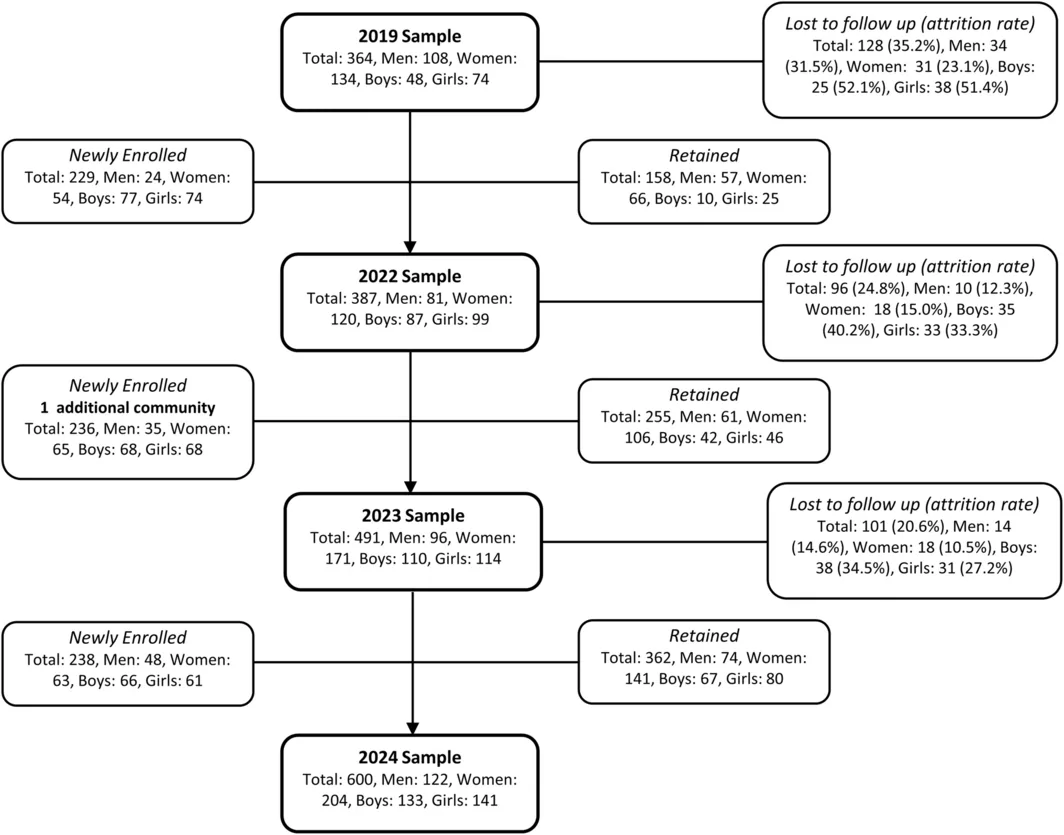
Daasanach human biology project enrollment, retention, and attrition from 2019 to 2024 before the start of the summer 2025 field season.

### 2.2 Study Design

The current study builds on the previous longitudinal study by expanding the sample size, focusing on adults, adding data collection during the rainy season, assessing a natural experiment, and collecting biomarker measurements of kidney health. The target sample size is > 600 adults in 450 households across 12 villages. The study is ongoing and is planned for 6 waves of data collection over 3 years, followed by an attrition survey. The study is structured around seasonal visits, once in the dry season (June–July) and once in the longer of two annual wet seasons (March–May) to capture seasonal changes in water salinity and health outcomes.

### 2.3 Sampling and Enrollment

The 7 original communities in the longitudinal study were selected to capture variation in drinking water salinity and distance to the main market town with 2 communities gaining access to desalinated water. For the new longitudinal study, in addition to the original 7 communities, 5 communities will be purposively selected to include 2 communities with access to the desalinated water system, 1 community that is on Lake Turkana, and 2 communities outside the main market town. Therefore, households in 4 out of the 12 communities will have access to desalinated water, while the other 8 will have variation in salinity levels.

For initial enrollment in the study, we will use the WHO random walk sampling scheme in large communities, selecting the 3rd household from the community center with help from the community health workers to have ≥ 25 independent households per community. This sampling strategy reduces familial clusters. If the adult is not home, the neighbor will be invited to participate. We will use a representative stratified sampling strategy for all components to attempt a 50% male, 50% female, and 50% between 18 and 45 years, and 50% 45+ year split, referring to the demographic census to update our sampling as enrollment and selection is ongoing. For follow‐up waves, we will re‐contact the initial households.

Due to cost and logistics, we will conduct a doubly‐labeled water (DLW) component of the study to measure water turnover and energy expenditure with 25% of the sample (*n* = 150). For each of the 3 years, there will be a new (*n* = 50) subsample with repeated observations in the dry and wet season.

Before collecting data for a particular community, we will first meet with the Illeret Health Clinic, a local public health advisor, and village elders within the study communities. In coordination with community health volunteers, we will schedule appointments with study participants to gain consent. The following interview as well as other health data collection procedures are performed at a community‐specific site, such as a clinic, school, or church.

### 2.4 Materials Used for Data Collection

#### 2.4.1 Household Surveys

Trained Daasanach translators will conduct a 35–60‐min interview with participants on the measures described below and assist with testing and sampling drinking water and secondary water sources cited in surveys.

#### 2.4.2 Laboratory and Point‐of‐Care Testing

Several biomarkers will be measured in the field as well as in the laboratory using urine and fingerprick blood samples following human biology standards (Madimenos et al. 2022). In‐field measurements will rely on POC devices and urine test strips that provide results within seconds to minutes. Kidney function will be measured using a POC device (Novamax Pro Creatinine/eGFR) which will calculate estimated glomerular filtration rate (eGFR) using whole blood. A lipid panel will be measured using CardioCheck to measure HDL/LDL and triglycerides cholesterol. Urinalysis will be conducted on spot urine samples for all adult participants for albumin, creatinine, and protein with adjustment for time of day using Accutest Microalbumin/Creatinine 2–1 Urine Reagent Strips and Siemens Multistix 10 strips. Urine specific gravity, a measure of hydration status, will be measured using refractometers (Atago Pen). Biological samples, like urine and dried blood spots, will be kept frozen in the field, transported frozen to be stored in a −20°C freezer at the National Museums of Kenya, and then shipped frozen to Penn State University and placed in a −80°C freezer until they undergo laboratory analysis.

#### 2.4.3 Anthropometrics and Clinical Measurements

Height will be measured using a portable Seca standing stadiometer, placed on a hard, flat surface. Using a Tanita BF‐680W bioelectronic impedance scale, we will measure weight (to the nearest 0.1 kg), body fat percentage, and percent total body water. We will measure the sum of tricep, bicep, suprailiac, and sub‐scapular skinfolds with calipers and use Lohman's equations to calculate body composition (Lohman et al. 1988). Blood pressure will be measured using an Omron 10 series wireless blood pressure device.

#### 2.4.4 Environmental Measurements

Environmental measurements will include temperature and water quality measurements taken with field devices. Temperature and related measures will be quantified using the Kestrel 5400 Heat Stress tracker. This device measures ambient temperature, wet bulb globe temperature (WBGT), and heat index. Water quality will be assessed in‐field using a YSI ProDSS Multi‐Parameter Water Quality Meter. This device will measure salinity, turbidity, conductivity, pH, and temperature of water taken from water sources cited in the household survey. Additionally, we will transport 500 mL water samples to assess ionic composition at the CropNuts Laboratory for analyses in Nairobi, Kenya.

#### 2.4.5 Geographic Data

Geographic data will be collected using a Garmin InReach device. We will collect GPS points of water sources as well as individual households to measure distance from household to water source. Additionally, GPS tracking will be collected using CatLog 1 GPS devices (Perthold Engineering), which periodically records coordinates so that movement and distance can be measured.

### 2.5 Measures

A summary of measures across aims is presented in Table 2, below we describe each measure.

**TABLE 2:** Summary of aims and measures for the longitudinal study.

Aim 1: Water salinity and hydration				
Variable	Use	Timepoints, sample size	Method	Aims
Water salinity (chem. and phys. characteristics)	Exposure	36, variable (*n* ~ 36)	In‐field water samples	1–3
Ionic composition (NaCl) index (% for all sources)	Exposure	6, variable (*n* ~ 36)	Transported water samples	1–3
Heat stress (e.g., WBGT)	Exposure	6, *n* = 600	In field Kestrel	1–3
Heat illness history*	Moderator	6, *n* = 600	Survey	1–3
Urine specific gravity	Outcome	6, *n* = 600	Urine sample	1
Urine osmolality*	Outcome	6, *n* = 600	Transported urine sample	1
Water Turnover (L/day)	Outcome	6, *n* = 150 (*n* = 50/year)	Subsample urine; (DLW)	1
Water insecurity	Outcome	6, *n* = 600	Survey	1
Thirst (perception)	Outcome	6, *n* = 600	Survey	1
Heavy metals*	Moderator	6, variable (*n* ~ 36)	Transported water samples	1‐3
Anthropometrics (eg, Wt, BMI, %BF)	Controls	6, *n* = 600	In‐field measures	1–3
Socio‐ecol. (e.g., SES, food insec, tobacco)	Controls	6, *n* = 600	Survey	1–3
Phys. activity (axivity)	Control	6, *n* = 150	Subsample DLW	1
Long‐term GPS/mobility	Control	6, *n* = 150	Subsample DLW	1
Sex	Moderator	6, *n* = 600	Survey	1–3
Age	Moderator	6, *n* = 600	Survey	1–3
Reproductive status (e.g., pregnant, lactating, menstruating)	Moderator	6, *n* = 600	Survey	1–3
Seasonality	Exposure	6, *n* = 600	Survey	1–3
Time (year)	Control	6, *n* = 600	Survey	1–3
Desalinated water (y/n)*	Exposure	6, *n* = 600	Survey	1–3
Diet (water, milk, salt)	Control	6, *n* = 600	Survey FFQ	1–3
Aim 2: Water salinity and kidney health				
Creatinine and eGFR*	Outcome	6, *n* = 600	POC fingerpick	2, 3
Albuminuria	Outcome	6, *n* = 600	In‐field urinalysis	2, 3
Proteinuria*	Outcome	6, *n* = 600		2
NGAL*	Outcome	6, *n* = 600	Transported urine sample	2
Uromodulin*	Outcome	6, *n* = 600		2
Aim 3: Water salinity and BP and hypertension and mediation				
Systolic and diastolic BP	Outcome	6, *n* = 600	In‐field cuff	3
Hypertension Stage 1/2	Outcome	6, *n* = 600		3
Hypertension	Mediator	6, *n* = 600		3/Hypothesis 4

*Note:* *expanded from parent study.

#### 2.5.1 Hydration and Water Insecurity Outcomes

##### 2.5.1.1 Urine Specific Gravity

Urinary biomarkers of hydration status provide critical data on how well people are meeting their water needs. While they normally lag ~30 min from blood biomarkers of hydration status, urinary biomarkers are non‐invasive and field friendly (Oppliger et al. 2005). Urine specific gravity (USG) is the best field‐friendly method and will be measured using pen refractometry on spot urine samples (Wutich et al. 2020). Urine samples will be collected at the time ambient weather conditions are measured prior to the survey, covered and placed in the shade for 15 min prior to measurement. Measurements will be conducted in triplicate with the average used in analyses. Values greater than 1.020 reflect dehydration.

##### 2.5.1.2 Urine Osmolality

Once samples are frozen and shipped to Penn State University, they can be measured for urine osmolality in duplicate on an Osmo1 osmometer using freezing point depression osmometry as a second hydration biomarker when necessary (Wutich et al. 2020).

##### 2.5.1.3 Water Turnover and Energy Expenditure

Using the doubly labeled water (DLW) method a subsample of individuals will ingest a dose of isotope enriched water (10% H_2_‐^18^O, 6% ^2^H_2_‐O, 1.25 g per kg body weight) for measurement of water turnover (L/day) and daily energy expenditure (kcal/d) (Speakman et al. 2021). The pre‐survey urine sample will be used to establish isotope levels at baseline, and additional urine samples at 6 h post‐dose and on Day 3 and Day 7 post‐dose will be used to determine the dilution spaces and depletion rates of ^2^H and ^18^O via the slope‐intercept method. Urine (5ml) samples will be stored frozen (−20°C) in the field and transported back to the U.S. where they will be stored at −80°C prior to centrifuge filtering and isotope analysis using laser‐based mass spectrometry (ABB ICOS) (Wutich et al. 2020). Isotope depletion rates will be used to calculate mean water turnover and daily energy expenditure over the 7‐day measurement period using validated equations (Speakman et al. 2021). Using an individual's drinking water salinity and total daily water turnover, we will estimate salt intake for comparison to dietary recall.

##### 2.5.1.4 Water Insecurity

Water insecurity will be assessed with each household head at an individual level using the validated Household Water Insecurity Experiences (HWISE) scale (Young et al. 2019), which has been translated and used in this site. The 12 questions on this scale ask about the frequency in the prior four weeks of experiencing: (A) emotional distress (worry, anger, or shame) related to water situation; (B) disruptions in daily life due to water problems (had to change what was eaten; unable to wash clothes, body, or hands; had to change plans); (C) physiological discomfort due to not having enough water (unable to drink as much water as desired or going to bed thirsty); or (D) interruptions in the water supply or no usable water in the household whatsoever. The score for this scale ranges from 0 to 36.

##### 2.5.1.5 Perceived Thirst

We will assess three measures of perceived thirst: current thirst, pleasantness of drinking water, and perception of dry mouth using locally‐adapted visual analog scales (Phillips et al. 1984) which have been used in this setting previously (Rosinger et al. 2022). For each of the questions, participants are asked to rate their current thirst on a scale from 1 to 10. We will then provide participants with clean water to drink and proceed with the survey.

#### 2.5.2 Kidney Health Outcomes

##### 2.5.2.1 Creatinine and eGFR

Creatinine will be measured using a fingerstick capillary blood test in a POC device (NovaMax Pro Creatinine/eGFR) that uses the CKD‐EPI 2021 equation to estimate GFR (eGFR) (Miller et al. 2021). This POC device has excellent correlation with laboratory methods (*r* = 0.99) (Gbinigie et al. 2015; Lee‐Lewandrowski et al. 2012; Naugler et al. 2014). We will define kidney function as: Stage 1 normal or high GFR (GFR > 90 mL/min); Stage 2 Mild CKD (GFR = 60–89 mL/min); Stage 3A Moderate CKD (GFR = 45–59 mL/min); Stage 3B Moderate CKD (GFR = 30–44 mL/min); Stage 4 Severe CKD (GFR = 15–29 mL/min); Stage 5 End Stage CKD (GFR < 15mL/min) (Miller et al. 2021). Urinalysis will be conducted on spot urine samples for all participants for albumin, creatinine, and protein with adjustment for time of day.

##### 2.5.2.2 Albuminuria

Urine test strips will be used to measure albuminuria (defined as ≥ 30 mg/g and > 300 mg/g) (Accutest Microalbumin/Creatinine 2‐1 Urine Reagent Strips has 92% and 86% accuracy [agreement with Bayer Clinitek Microalbumin] for albumin and creatinine, and 97% and 92% precision).

##### 2.5.2.3 Proteinuria and Hematuria

Proteinuria generally signifies damage to the glomerular filtration barrier. Less commonly it can result from damage to the tubules. Protein is not detected in the urine of healthy kidneys. A qualitative assessment of proteinuria (none, mild 1+, heavy 2+) will be calculated from urine (Hemmelgarn et al. 2010). Similarly, hematuria or the presence of blood will be captured from the urine dipsticks (none, trace, mild 1+, heavy 2+) (Siemens Multistix 10 strips).

##### 2.5.2.4 Neutrolphil Gelatinase‐Associated Lipocalin (NGAL)

Neutrolphil Gelatinase‐Associated Lipocalin (NGAL) is a biomarker used to identify acute kidney injury (AKI). NGAL uses the following criteria: 50–149 ng/mL for equivocal AKI risk and repeat measurement indicated if clinically justified; 150–300 ng/mL for moderate AKI risk and test result has high sensitivity/moderate specificity; > 300 ng/mL for high AKI risk and test result has high specificity. This biomarker will be measured using R&D Quantikine enzyme‐linked immunosorbent assays (ELISAs) (Kift et al. 2013) and indexed to urine creatinine.

##### 2.5.2.5 Uromodulin

Uromodulin (i.e., Tamm‐Horsfall protein) is a 95‐kDa glycoprotein produced by the thick ascending limb of the loop of Henle and early distal convoluted tubule (Schaeffer et al. 2021; Youhanna et al. 2013; Zhang et al. 2018). It is an established kidney tubule injury biomarker for CKD in the general population. Urinary uromodulin is the most plentiful urinary protein (20–70 mg excreted daily) in healthy adults and decreases when renal function declines and will also be assessed using R&D ELISA (Nqebelele et al. 2019).

#### 2.5.3 Systolic/Diastolic Blood Pressure and Hypertension

Using the Omron 10 series wireless device, systolic and diastolic blood pressure and pulse will be measured twice per person in the field for each survey wave with the average value used (James and Gerber 2018). The two measurements will be taken while the participant is sitting post‐interview. We will carefully assess BP using standard guidelines to ensure accurate measurement (Unger et al. 2020). This includes having the participant comfortably seated for 5 min prior to measurement, legs uncrossed and feet flat on the ground while the back and arm are supported, removal of clothing that covers the location of the cuff, arm/cuff at heart level, no talking during the assessment, avoidance of exercise, smoking, and tea/coffee for at least 30 min prior to assessment, ensuring the participant has emptied their bladder, and repeat measurement separated by 1–2 min. Classifications of hypertension are Stage 1 (≥ 130|80 mmHg) and 2 (≥ 140|90 mmHg) (Unger et al. 2020).

#### 2.5.4 Exposures

##### 2.5.4.1 Water Quality Measures: Salinity and Ionic Composition

The primary exposure in this study is the sodium‐chloride concentration of water because these salts are associated with increased BP and kidney damage. As many potential elements may be associated with our outcomes of interest, water quality will be assessed for different salts, heavy metals, and overall salinity. Specifically, other salts such as potassium (K), magnesium (Mg), and calcium (Ca) will be controlled for in models because they are seen as cardioprotective and may buffer the undesirable effects of sodium and chloride (Rosinger et al. 2025). In prior work, we demonstrated that the mean sodium‐chloride concentration in drinking water sources was 163 mg/L while the average calcium/potassium/magnesium concentration was 45 mg/L (Table 3) (Rosinger et al. 2025).

**TABLE 3:** Preliminary water quality analysis stratified by drinking or non‐drinking water sources sampled across 2022 and 2023.

	Water source		
	Drinking^a^ (*n* = 25) mean (SD)	Primarily non‐drinking^b^ (*n* = 6) mean (SD)	Desalinated (*n* = 2)^c^ mean (SD)
Total dissolved solids (mg/L)	430.5 (214.0)	1948.3 (733.2)	128.5 (0.7)
Total salinity (mg/L)^d^	378.0 (232.5)	1873.3 (478.2)	110 (14.1)
Sodium (mg/L)	122.5 (84.8)	621.7 (231.2)	42.4 (5.2)
Chloride (mg/L)	46.1 (44.5)	621.3 (370.5)	46.7 (1.9)
NaCl (mg/L)	168.6 (126.3)	1243.0 (580.9)	89 (7.1)
Calcium (mg/L)	19.4 (12.6)	15.0 (9.6)	0.1 (0.1)
Magnesium (mg/L)	5.8 (5.3)	7.9 (6.9)	0.5 (0.70)
Potassium (mg/L)	7.1 (6.6)	17.3 (9.9)	1.4 (1.6)
Ca/Mg/K (mg/L)	32.3 (17.9)	40.2 (15.2)	2 (1.0)

^a^ Drinking water comes from hand‐dug wells and shallow well‐standpipes. Non‐drinking water comprises borehole standpipes and Lake Turkana.

^b^ Dry riverbed hand‐dug well represents twelve different hand‐dug well locations, of which six were sampled both in 2022 and 2023.

^c^ The same desalinated source was sampled across both years.

^d^ Sampled with YSIS ProDSS water quality meter directly in the field. All other analyses measured at CropNuts Laboratory using atomic absorption spectrophotometry. Table modified from Rosinger et al. (2025).

We will collect water samples to be analyzed in a lab for their ionic composition, including salinity concentrations and heavy metals using atomic absorption spectrophotometry (Omer 2019). Specifically, we will measure for pH, total dissolved solids, electrical conductivity, turbidity, molybdenum, arsenic, ammonium, cadmium, calcium, chromium, magnesium, cobalt, potassium, lead, sodium, mercury, nitrate‐nitrogen, nickel, nitrates, selenium, zinc, phosphorous, copper, sulfur, boron, sulfate, aluminum, iron, manganese, chloride, bicarbonate, fluoride, hardness (CaCO_3_), silicon, and silicon dioxide (silica). In addition, we will measure total water salinity in the field using a portable field device (ProDSS) to provide real‐time results with community members.

We will account for multiple water source use (including during travel) by creating a water salinity index dose estimating an individual's exposure to salinity at the time of biomarkers based on all water sources they report ingesting and frequency of use.

##### 2.5.4.2 Heat Stress

At the time of surveys, we will record (1) wet bulb globe temperature (WBGT), a weighted metric combining the effects of temperature, humidity, and solar radiation, and impacted by air movement and (2) heat index (HI) using a Kestrel 5400 Heat stress tracker. WBGT is a physical index of the environment whereas HI is a perceptual index. We will use categorical thresholds associated with both WBGT and HI that can be used to predict heat stress compensability under activities of daily living (Vecellio et al. 2022). For higher metabolic rates associated with daytime activity, WBGT will be used to categorize heat strain conditions based on current National Institute for Occupational Safety and Health (NIOSH) guidelines. We will use combined time‐weighted activity estimates with WBGT and HI to assess overall thermal load.

##### 2.5.4.3 Heat Illness History

We will assess past experiences with heat illness including heat stroke (timing, severity—explained through translators) modified from the NIOSH health hazard evaluation of heat stress (HETA‐2012) in our survey (Centers for Disease Control and Prevention 2018).

##### 2.5.4.4 Seasonality

This longitudinal study will take place during the dry and wet seasons. Seasonality, therefore, will serve as a dummy variable exposure in the analysis.

##### 2.5.4.5 Time

Year will be included in the analysis as controls as well as part of the multi‐level design of the analysis.

##### 2.5.4.6 Desalinated Water

Some community members who live close to the desalination system can retrieve this water free of cost. Currently, the desalinated water source comes from the Turkana Basin Institute, which has a borehole that it pipes to its reverse osmosis filtration system. Our analyses show that the borehole water has a NaCl concentration of 1324 mg/L (data not shown), which after desalination is reduced to ~89 mg/L (Table 3). Communities with access to desalinated water will be asked (yes/no) if they use this type of water and if so, how frequently and what quantity. In the analysis, this exposure variable will be used as an intent‐to‐treat effect.

#### 2.5.5 Other Measures

##### 2.5.5.1 Demographic

Basic demographic information, such as sex, education, age, and occupation will be collected in the household survey. Socio‐economic status (SES) will be assessed in several ways: we will measure reported income (Kenyan shillings earned in the last month) along with participant's livestock wealth (the summed market value of all goats, sheep, cattle, camels, and other livestock owned), self‐perceived social status measured by asking them to rank themselves on a MacArthur Ladder from 1 (worst off) to 10 (best off) relative to others in their community regarding income, education, and social status, and, for men, the number of wives they have (Giatti et al. 2012).

##### 2.5.5.2 Mobility

Mobility will be assessed through a question about the number of times they have moved in the prior season, with follow‐up questions about water sources used during travel. This will also allow us to know specific locations of all used sources (estimated *n* ~ 40) for water quality and GPS sampling.

##### 2.5.5.3 Self‐Reported Physical Health

Self‐reported health will be assessed using recall in the past 7 days for diarrhea (defined as three or more instances of loose or watery stool in a 24‐h period), headache, fever, and cough/other respiratory infections. Participants will also self‐report malaria infection in the previous year and if the infection was confirmed by a laboratory test. Chronic conditions, including diabetes, hypertension, kidney problems, and kidney stones, will be asked whether a health professional has ever told the participant they have the condition. Finally, regular medication use will also be collected via self‐report.

##### 2.5.5.4 Anthropometrics

Anthropometric measurements include height, weight, skinfolds, as well as body fat percentage. We measure height and weight so that we can calculate BMI and weight status categories. In this population, since mean BMIs are low, we will control for the underweight classification (BMI < 18.5 kg/m^2^) which may influence the outcomes.

##### 2.5.5.5 Physical Activity

Physical activity will be assessed via a modified Global Physical Activity Questionnaire of all participants. We will also assess physical activity directly with Axivity A3 watches among the DLW subsample to acquire 7 days of activity data to overlay water turnover and energy expenditure.

##### 2.5.5.6 Food Insecurity

Food insecurity will be measured with the validated household food insecurity access scale (HFIAS) to assess experiences with insufficient access to food (Coates et al. 2007). This module in our survey consists of twelve questions surrounding anxiety, quantity, access, and experiences concerning food consumption in the household. We also ask about salt consumption as well as food aid. The HFIAS instrument is a score that ranges from 0 to 27.

##### 2.5.5.7 Mental Health

Mental health will be measured using two measures: perceived stress and anxiety/depression. Psychosocial stress will be measured using Cohen's Perceived Stress Scale‐4 (PSS‐4). The PSS‐4 is a 4‐question module that asks about how events in a person's life are perceived in terms of their ability to manage them (Cohen et al. 1983). It asks about how much control an individual perceives they have over events occurring in their life and about their confidence to manage such events. Anxiety/depression will be assessed with the modified for African contexts Patient Health Questionnaire‐9 (PHQ‐9) (Brewis et al. 2021). The PHQ‐9 scale is a verified questionnaire containing nine questions that correspond to depressive symptoms and has been validated in multiple contexts such as East Africa (Gelaye et al. 2013; Monahan et al. 2009). The modified PHQ‐9 replaces one question about suicide with two questions about anxiety (Brewis et al. 2021). We do this due to cultural sensitivity issues around topics of suicidal ideation.

##### 2.5.5.8 Conflict

Using the HWISE style question, respondents are asked whether they have experienced conflict with their spouse, and separately with neighbors due to any water issues in the last 4 weeks. The responses vary from 0 (Never) to 3 (Often or always).

##### 2.5.5.9 Long‐Term GPS/Mobility

We will measure mobility patterns and heat exposure during daily activities using customized long‐term CatLog1 GPS tracking units (Perthold Engineering) to record location and ambient temperature at 5‐s epochs to track mobility. Collecting location data over time will allow us to calculate distance traveled as well as heat exposure for the DLW subsample and overlay it with physical activity data.

##### 2.5.5.10 Reproductive Status

At each survey wave, adult female participants will be asked to self‐report their current reproductive status. They will be asked if they are pregnant, and if so for how many months; if they are lactating, and if so for how many months. Finally, they will be asked whether they are currently menstruating or not.

##### 2.5.5.11 Diet: Food Frequency Questionnaire

We will ask about the frequency of consuming certain staple foods to assess food, water, and beverage intake, including milk. Twenty‐one food items, such as cow meat, fish, *ugali* (a thick, cornmeal porridge), and cooking oil are listed, and the respondent provides the frequency they consume these items from 0 times/week, 1–3 times/week, 4–6 times/week, 1 time/day, to 2+ times/day. We will also calculate total salt ingestion—and estimate it from drinking water, tea, cooked foods, and milk. This food frequency questionnaire was developed in coordination with community members with a deep knowledge of local diets and culture. This ensured that we included appropriate food items to represent Daasanach diets accurately.

## 3 Data Management and Analytical Plan

After each data collection phase, we will perform double data entry into an electronic database. The longitudinal structure of the database will require the use of regression models that account for repeated observations over time. The aims detailed above will be analyzed individually using multi‐level regression models.

For Aim 1, we seek to assess how variation in drinking water salinity relates to hydration status, water turnover, and water insecurity. Four different levels are specified where years are nested in people that are nested in households which are nested in communities. Outcomes are water salinity, hydration status, water insecurity, and thirst ($y_{\text{ijkl}}$). We specify random variance components of the intercept at each level ($u_{000l}$, $v_{00\mathrm{kl}}$, and $w_{0\mathrm{jkl}}$); covariate effects (e.g., heat stress) omitted for brevity.

$$y_{\text{ijkl}}=\gamma_{0000}+\gamma_{1000}S_{\text{ijkl}}+u_{000l}+v_{00\mathrm{kl}}+w_{0\mathrm{jkl}}+e_{\text{ijkl}}$$

For the salinity model, season ($S_{\text{ijkl}}$: wet versus dry) is the primary predictor, whereas in all further models salinity index is the primary predictor with season acting as an adjustment. With 3 years of data, and *n* = 600 from 1.5 people per household, 33 households per community, and 12 communities, a conservative estimate for the minimal detectable effect size (MDE) at 80% power and 5% alpha as computed by Optimal Design (Spybrook et al. 2011) is *d* = 0.318, or less than one third of a standard deviation. That is, we will have power to detect moderate to small effects of season and salinity. Based on our prior research, we expect effects to be larger than the MDE (Bethancourt et al. 2021; Mcgrosky et al. 2022; Rosinger et al. 2022).

For Aim 1, in addition to the above analysis on the full sample, we will have a subsample (*N* = 150) from whom DLW will be collected twice per year to assess water turnover. For this subsample, a similar modeling approach is taken; however, because each person is measured in only 1 year rather than in all three, the lowest level of nesting (i.e., year) is removed.

For Aim 2, we rely on a similar modeling approach as described in Aim 1. It explores how water salinity impacts kidney function over time. We account for repeated measures, time, and nesting in the data. Instead of season as the primary predictor in this model, salinity index is used. The outcome will include repeated measures of kidney function and injury markers. The equation for this model is similar to Aim 1 but uses the salinity index variable ($N_{\text{ijkl}}$) as the primary predictor instead of season ($S_{\text{ijkl}}$) which remains but merely as a covariate. For brevity in the equation, we have omitted explicit notation of all the included covariate effects (e.g., socio‐ecologic stressors, heat stress, SES, etc.).

$$y_{\text{ijkl}}=\gamma_{0000}+\gamma_{1000}S_{\text{ijkl}}+\gamma_{2000}N_{\text{ijkl}}+u_{000l}+v_{00\mathrm{kl}}+w_{0\mathrm{jkl}}+e_{\text{ijkl}}$$

Beyond this simple model for the effect of salinity, we will add several covariates that capitalize on several features of these data. First, there is a natural experiment that provides some of the communities with desalinated water. We will create a dummy‐coded indicator for adults within these communities who receive desalinated water and add this to the model as an intent‐to‐treat effect. A similar simple approach will be used to incorporate this natural experiment into all the subsequent models.

For Aim 3, a similar approach is used to that of Aim 2. It explores how water salinity impacts systolic and diastolic BP and hypertension risk over time. It further explores how changes in BP affect kidney health. The modeling approach (and equation) to evaluate the effect of water salinity on BP is similar to Aim 2. This model accounts for both seasonal variation over time and longitudinal variation over years, the latter with the index *i* for years. We seek to test whether BP could mediate the relation between water salinity consumption and kidney damage. This mediation does not strictly depend on a prior statistically significant finding for the relation between kidney function and water salinity consumption. A mediation effect can be present regardless. To explore this, we fit multilevel models related to each of the paths in the mediation and test combined statistics from these models (Preacher et al. 2011).

Differences in vulnerability exist across age, gender, and reproductive health status. For this reason, models across all aims will control for these covariates. Other relevant covariates include dietary intake, milk consumption, heat stress (WBGT), physical activity, and socio‐ecological stressors. Age is a particularly important consideration since older populations are more vulnerable to dehydration during heat waves and (Kenney et al. 2014; Kenny et al. 2010; Semenza et al. 1996). Furthermore, pregnant women can be at greater risk of gestational hypertension when exposed to drinking water salinity (Scheelbeek et al. 2017), and lactating women have a higher risk of dehydration in hot environments (Rosinger 2015). Through collecting this data, we can specify variables as continuous, categorical, as well as split analyses based on sub‐groups to understand drinking water salinity effects on health.

### 3.1 Data Linkage With Prior Waves of Daasanach Human Biology Project

An additional possibility with this ongoing expanded longitudinal study is linking survey participants to prior survey waves from 2019 to 2024. We will use anonymous id values generated for each individual to combine with previous data collection phases. Two datasets will thus be created, one associated with this current study and another attached to prior survey waves.

## 4 Community Engagement

Through long‐term work in the region, we have developed a strong relationship with local Daasanach communities. We base this project on key community concerns (water salinity and water insecurity) that emerged from several years of discussions, focus groups, and interviews prior to the implementation and during the Daasanach Human Biology Project longitudinal study. This project is supported by current on‐the‐ground relationships with the local Illeret Health Clinic and multi‐level national and regional support from the National Museums of Kenya and the Kenya Medical Research Institute (including the Kenya Ministry of Health).

The Daasanach Human Biology Project works closely with various institutions in Nairobi and Illeret during all phases of data collection. We also will maintain and strengthen our relationship with local groups in Illeret. First, we will continue to work with communities and local institutions to build and maintain rapport. The Illeret Health Clinic will be one site of data collection for the project. We will communicate research plans to and share results with the clinic as well as donate supplies that could be useful. Likewise, as we rely on other local institutions, such as schools, as sites of data collection, we will work with administrators to donate supplies that can be useful for teachers and students. Translators are hired from Daasanach communities in Illeret. This helps build capacity as well as confer data collection and translation skills to community members working on the project. We will share results with local officials and inform them of our future plans. For instance, we met with officials in Marsabit County as well as from the North Horr sub‐county to present results over a two‐day workshop as well as describe plans for this study in 2025. Finally, Daasanach participants will benefit from this study by receiving immediate results from clinical and point‐of‐care measurements, like blood pressure, eGFR, and hydration status. These results will be communicated and explained to participants with the help of translators. If a measurement indicates a health concern for a participant, we will refer them to the health clinic, thereby fostering an ongoing relationship between community members and local institutions.

## 5 Discussion

The Daasanach Human Biology Project provides a unique opportunity to understand how emerging environmental and social stressors, including drinking water salinity and heat stress, affect human health with implications for populations around the world. These topics have been largely under‐explored when it comes to human biology research among pastoralists (Fujita et al. 2025; Galvin 1992; Nathan et al. 1996; Pike et al. 2010; Sayre et al. 2019; Straight et al. 2025). Water salinity is an emerging human health risk exacerbated by several climatic and anthropogenic processes. Yet, little is known about how water salinity impacts kidney health, hydration, and cardiovascular health. This longitudinal study compares communities exposed to water salinity to those with access to desalinated water in a hot‐arid environment. Using an intensive longitudinal design leveraging a natural experiment with variation in water salinity while adjusting for co‐exposures can help identify dose‐response relationships on hydration, kidney function, and blood pressure for populations exposed to salinity. By testing how upstream intervention points, like water desalination, can mitigate these significant public health concerns, results can be translatable for populations exposed to high water salinity levels. Results from this study can inform worldwide recommendations for health standards related to water salinity levels, since currently none exist beyond palatability.

Attrition is a potential limitation of longitudinal studies. We will mitigate and address attrition pre‐emptively, during each wave, and post‐survey. Pre‐emptively, our long‐term work and employment of community members can increase participation and trust. During the survey, the majority of data are collected in the same day to avoid attrition within a study wave. Second, we will use refreshment samples in each wave with individuals similar in characteristics to those missing to maintain sample size (Deng et al. 2013). Following the 6 waves, we will re‐visit attriters who moved outside the study communities. We will conduct attrition analysis to assess whether those who left systematically differed from those who remained. Our analytic strategy of multilevel models handles attrition well (Mallinckrodt et al. 2001). In the baseline survey, we will collect data that may predict who is likely to move (e.g., based on wealth or mobility), which we can include in models as covariates to reduce bias (Enders 2010). We will also use multiple imputation methods to deal with missing data (Twisk and de Vente 2002). To account for potential attrition bias, we will conduct sensitivity analyses and control for the number of survey waves in which an individual was present. If attrition is related to health outcomes, the addition of the count variable should change the parameter estimate.

## Author Contributions

A.Y.R. and A.J.R. prepared the main manuscript. All authors contributed to editing and reviewing the manuscript.

## Funding

Funding for this study comes from the National Institute of Environmental Health Science (NIEHS) R01 Award titled “Drinking water salinity in a hot‐dry environment: Hydration, kidney function, and blood pressure” (R01ES035402), and support from the National Institute on Aging (NIA) Center Grant to Duke University Population Research Institute (P30 AG034424) and NIH’s Eunice Kennedy Shriver National Institute of Child Health and Human Development (NICHD) (P2CHD041025). This work was also funded by Penn State University's Institute for Energy and the Environment, Social Science Research Institute, and College of Health and Human Development. It was supported by the National Science Foundation (#1924322 and #1852406). The content is solely the responsibility of the authors and does not necessarily represent the official views of the National Institutes of Health.

## Disclosure

No artificial intelligence (AI) tools were used at any point in manuscript preparation.

## Ethics Statement

This study has received ethical approval from the Pennsylvania State University Institutional Review Board (STUDY00009589), the Kenya Medical Research Institute (KEMRI/SERU/CVR/003/3739), the National Commission for Science, Technology and Innovation, the Marsabit County Government Ministry of Health, and Daasanach elders. Adult participants will be informed of the components and risks of the study, and all will provide oral or written consent (via signature or fingerprint in the case of illiteracy) with the assistance of Daasanach language translators. We will also share results from urinalysis and point‐of‐care (POC) devices with participants upon finishing each interview. Since certain biomarker results are ready within seconds to minutes, we will use this information to create a notecard that participants can collect as well as discuss with project leads for interpretation of results.

## Conflicts of Interest

The authors declare no conflicts of interest.

## Data Availability

All data are presented in this manuscript. Data generated from this study will be posted once the full study is complete.
